# Tracking human interactions with a commercially-available robot over multiple days

**DOI:** 10.12688/openreseurope.14824.1

**Published:** 2022-08-16

**Authors:** Ruud Hortensius, Bishakha Chaudhury, Martin Hoffmann, Emily Cross

**Affiliations:** 1 Institute of Neuroscience and Psychology, University of Glasgow, Glasgow, UK; 2 Department of Psychology, Utrecht University, Utrecht, The Netherlands; 3 Department of Computer Science, Humboldt University Berlin, Berlin, Germany; 4 Department of Cognitive Science, Macquarie University, Sydney, Australia; 5 MARCS Institute for Brain, Behaviour and Development, Western Sydney University, Sydney, Australia

**Keywords:** human-robot interaction, social cognition, social robotics, robotic platform, embodied interaction

## Abstract

**Background:** As research examining human-robot interaction moves from the laboratory to the real world, studies seeking to examine how people interact with robots face the question of which robotic platform to employ to collect data *in situ*. To facilitate the study of a broad range of individuals, from children to clinical populations, across diverse environments, from homes to schools, a robust, reproducible, low-cost and easy-to-use robotic platform is needed.

**Methods:** We describe how a commercially available off-the-shelf robot, Cozmo, can be used to study embodied human-robot interactions in a wide variety of settings, including the user’s home. We describe the steps required to use this affordable and flexible platform for longitudinal human-robot interaction studies. First, we outline the technical specifications and requirements of this platform and accessories. We then show how log files containing detailed data on the human-robot interaction can be collected and extracted. Finally, we detail the types of information that can be retrieved from these data.

**Results:** We present findings from a validation that mapped the behavioural repertoire of the Cozmo robot and introduce an accompanying interactive emotion classification tool to use with this robot. This tool combined with the data extracted from the log files can provide the necessary details to understand the psychological consequences of long-term interactions.

**Conclusions:** This low-cost robotic platform has the potential to provide the field with a variety of valuable new possibilities to study the social cognitive processes underlying human-robot interactions within and beyond the research laboratory, which are user-driven and unconstrained in both time and place.

## Introduction

Social robots offer vast new opportunities for our fundamental understanding of human psychology and applications in education, healthcare, social interaction, and other domains ^1– 4^. Embodied social interactions between humans and robots are the gold standard for the field of social robotics. They also remain one of the core challenges for the field, given difficulties with formal descriptions and predictions of social cues and dynamics across situations and norms ^5^. While this challenge has mainly been described from the perspective of the robotic platform (e.g., social signal processing, natural language processing), a critical point is that few studies examine truly embodied social interactions between humans and robots across a variety of environments, from controlled laboratory contexts to the real world. Running these studies is time-consuming, expensive, labour-intensive, and has mostly been limited to a few well-equipped laboratories that have the financial and computational resources to acquire one or more robotic platforms that allow for these social interactions. Here, we describe how to utilise a low-cost, commercially available and easy-to-use robotic platform, Cozmo (Digital Dream Lab, USA; https://www.digitaldreamlabs.com/pages/cozmo), to study embodied human-robot interactions (HRI) under a variety of conditions in a fully reproducible manner. 

 Researchers have several options at their disposal to approximate social interactions with robots that vary in level of complexity. The researcher might choose to sketch potential scenarios of interactions using vignettes or present images, animations, or films of robots on a screen or *via* virtual reality. Screen-based interactions are readily available and can enable researchers to examine a variety of social behaviours by proxy (e.g., emotion recognition, reward processing, collaboration). However, to better understand how these social behaviours manifest when interacting with robots in the real world, it is vital to perform HRI experiments with an embodied robotic agent (an agent that is physically present in the same room as the human user). Embodiment is critical for understanding the social cognitive processes underlying successful human-robot interactions ^4^. Researchers can use embodied agents to study one-off or repeated social interactions in or outside a laboratory. These interactions can be autonomous or use a wizard-of-oz methodology, where an experimenter controls the robot. When studying human interactions with embodied robots, the actual behaviour of the user and robot can be investigated in real time. To best capture all the layers that go into successful HRI (including emotional, cognitive, and behavioural), researchers are encouraged to study unrestricted, repeated, long-term, or longitudinal interactions with autonomous embodied robots ^6^. By doing so, this research should yield insights that will not only inform our understanding of human social cognition during interactions with robots ^7– 9^, but also the development of existing and new robotic platforms ^10^.

If researchers are to increasingly move towards unrestricted embodied interactions, then the urgent need for accessible embodied robotic platforms must be fulfilled. Fortunately, several commercially made robots are available to the community of HRI researchers. The NAO and Pepper robots (Aldebaran Robotics, France), iCub (Instituto Italiano di Tecnologia, Italy/RobotCub Consortium). and recently MiRo (Consequential Robotics, UK) are some well-known and frequently used robotic platforms that are targeted for HRI research. In recent years, several studies have also employed Cozmo (Digital Dream Lab, USA), a palm-sized robot, to study a variety of behaviours in the human user. Cozmo is a commercially made entertainment and educational robot targeted for children. Unlike the other robotic platforms mentioned here, Cozmo was not originally manufactured with the community of HRI researchers in mind when launched by the founding company Anki (USA). However, several research groups have started to use this robot in HRI studies. It has been successfully used in laboratories, schools, and public events, and with children and adult samples. Researchers have used it in experimental studies to measure perception and behaviour during one-off interactions ^11– 25^ and unrestricted and repeated interactions outside of the laboratory ^26, 27^. Together, these studies have investigated a variety of human behaviours, from empathy to emotion perception, and from social decision making and learning to the sense of agency in the human user. We believe that this platform will answer the call for a 1) low-cost, 2) easy-to-use, and 3) flexible robotic platform, both in terms of target population and setting, and that it will allow for 4) autonomous, 5) long-term or longitudinal, and 6) reproducible HRI studies. In the remainder of this article, we provide instructions on how to use Cozmo for human-robot interaction studies. After a detailed description of the technical specifications and potential of this platform, we present a step-by-step walkthrough on how to use this robotic platform for one-off, repeated, and longitudinal human-robot interaction studies. We show how researchers can record, collect, and extract log files from these interactions for off-line analysis. These log files contain detailed information on both the behaviour of the robot and human, thus providing insights into the quality and quantity of interaction. We describe what information can be retrieved from these logfiles and discuss the potential thereof. To facilitate in-depth analysis of the interaction we provide data on the behavioural repertoire of this robot. We present an interactive tool for emotion classification that will allow researchers in the field to get an informed understanding of not only the behaviour of the robot but also how the behaviour is perceived by the user.

## Methods

### Ethical statement

The study procedures used to develop the data extraction pipeline were approved by the Bangor Imaging Unit and the Bangor University School of Psychology Research Ethics Committee (protocol number: 2017-16209) and the Research Ethics Committee of the College of Science and Engineering at the University of Glasgow (protocol number: 300170226), and the emotion classification study procedure was approved by the Research Ethics Committee of the College of Science and Engineering at the University of Glasgow (protocol number: 300190004). This research project is not sponsored by, supported by, or affiliated in any manner with Anki or Digital Dream Lab. Active informed consent was obtained online from all eligible participants prior to inclusion in the study.

### Robotic platform architecture

***Robotic platform specifications and accessories***

**Figure 1.**
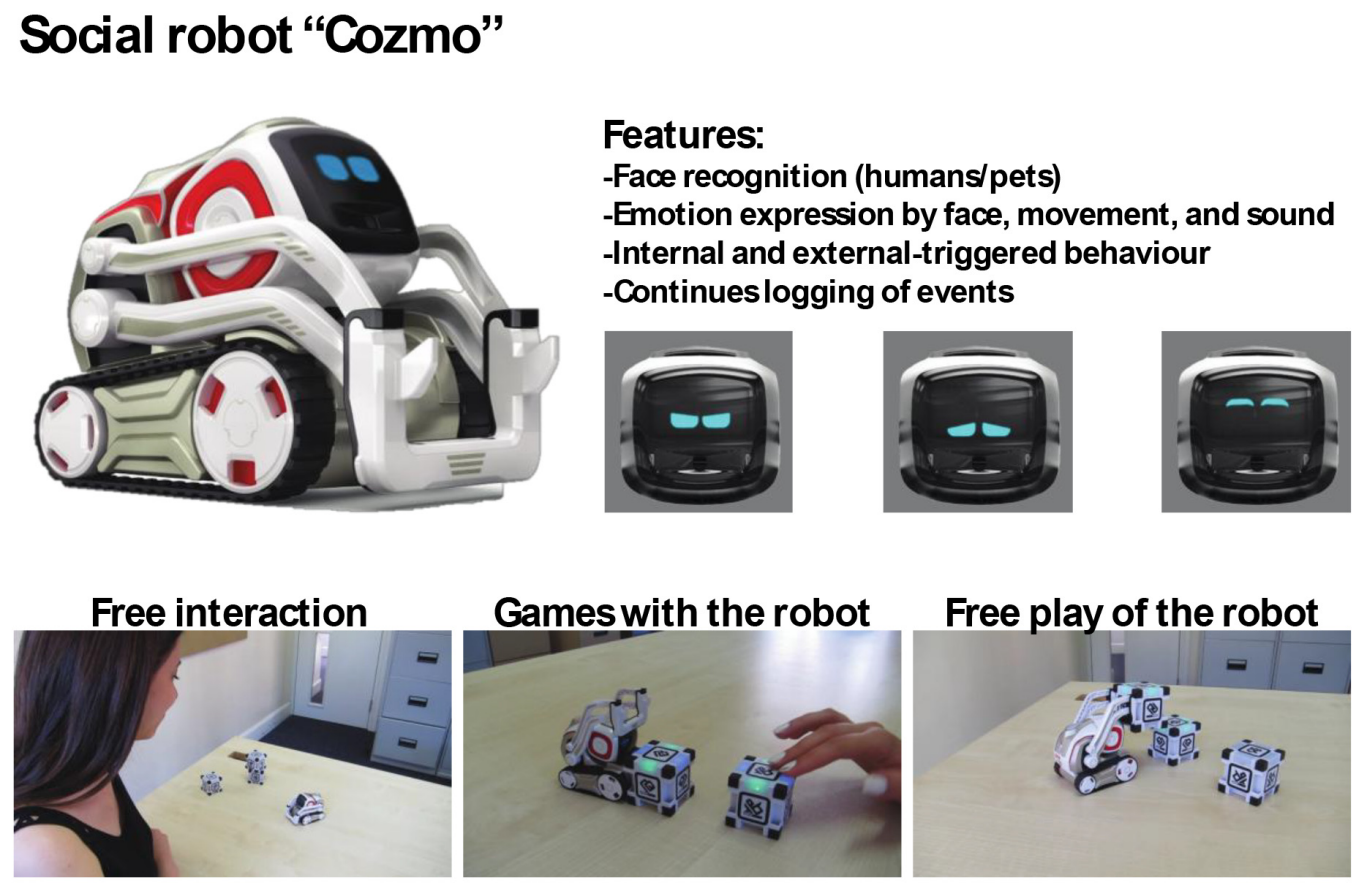
The social robot “Cozmo”. This robot can be used for embodied human-robot interactions in a wide variety of settings. Cozmo features face recognition of humans and pets, as well as the cubes, that are used in games and during free play of the robot. It uses facial, motion, and sound cues to communicate emotions based on an emotion engine that keeps track of internal- and external-triggered behaviour. The application associated with the robots allows for continues logging of events of the human-robot interaction. Photographs by Malte Heyen and Veronica Diveica.

Cozmo is a commercially produced social entertainment robot which comes with its own three light cubes and charger (Digital Dream Lab, USA). Cozmo was launched by an American company called Anki in 2016. Around April 2019 the company stopped the production of Cozmo as Anki shut down due to lack of investment. Towards the end of 2019, Anki’s assets were acquired by Digital Dream Lab and Cozmo and other Anki products are now back in production under the new brand. This robotic platform allows for people to interact with the robot in a diverse manner, ranging from free human-robot interaction to free play by the robot to specific games (e.g., memory games) ( Figure 1). The palm-sized robot used in this study (size: 5 x 7.25 x 10”) features a small head with a LED display (128 x 64 resolution) on which emotive eye animations are displayed. The eye animations are synchronised with corresponding sounds to make Cozmo’s responses more engaging. The Cozmo robot also features a low-resolution video graphics array (VGA) camera (30 FPS) through which it can detect its surroundings for navigating. It has four motors, >50 gears, and can move its head (up/down), fork (up/down), and tracks (forward/backward and left/right). It can recognise the light cubes, pets, human faces, and has the potential to recognise basic emotions in human users (for example, happy, sad, fearful, and surprised expressions). The light cubes feature markers that help Cozmo identify individual cubes. The emotion animations displayed by Cozmo are chosen by an emotion engine which keeps a measure of basic emotion parameters (e.g., happy, sad, annoyance). Cozmo interfaces with an application installed on a tablet or smartphone. Running this application, both internally (i.e., Cozmo’s emotion engine) and externally triggered reactions (i.e., interactions with the user, surroundings, pets, and other people) shape the interaction. The device running the application needs to be connected to Cozmo’s wifi. The Cozmo application is regularly updated using usual app update methods. These updates add new features and animations. The Cozmo application also gives access to a developer mode, where it can be used to run custom code written in Python (for more information see https://developer.anki.com/). In this mode, the Cozmo application needs to be connected to a computer with Python that also has the Cozmo programming packages ^28^ installed and running. The application then interfaces between the computer and the robot.

### Emotion engine

Cozmo reacts to its environment using internal behaviour transitions guided by its emotion engine. Environmental factors are picked up by Cozmo’s vision and artificial intelligence and generate reactions. Besides known and unknown humans, examples of environmental factors are pets, light cubes, and the detection of a cliff (i.e., an edge of a table or other surface). These environmental factors feedback values to Cozmo’s emotion engine to update basic emotion parameters (e.g., happy, sad, angry, bored). Based on the relative values of these parameters, Cozmo transitions between behaviours and animations displays. These reactions contribute to giving the robot an appearance of intent. Cozmo is built for instigating interaction. When it detects a pet, human or light cubes, it tries to engage with them. The emotion engine can guide Cozmo to automatically go into a mode where it is stacking its cubes or knocking over cubes without human intervention. Cozmo also invites humans to play games that the user may have unlocked during the course of their interaction. Whether the invitation is accepted or declined, Cozmo responds with an appropriate embodied emotional reaction. Game events or the lack of any external triggers (e.g., no user engagement with Cozmo for a sustained period of time) also influence the values of the emotion engine. The intensity of the emotion animation varies based on the context. For example, the reaction to losing an entire game would trigger an annoyance animation with higher intensity than when losing the first round of the game. The emotion animation displayed also feeds back values in the emotion engine. Every behaviour transition and animation display that is triggered by the emotion engine is logged by the app.

### Case study

For the initial study by Cross *et al.,* ^26^, for which we monitored participants’ interactions with Cozmo, we sent a Cozmo robot home with each participant for five days. We provided the participant with a tablet as well as the robot. This allowed us to lock the version of the Cozmo app we were using for the study and prevent participants from using other apps. The auto updates of the Cozmo app improve it and add new animations, games, and features. Since we wanted all participants to have a consistent view/version of Cozmo over the period of the study, we froze the version and prevented such updates. Due to cost and memory considerations, we chose Lenovo Android Tablets. We used Lenovo Tab 7 (1GB RAM + 8GB storage) and Lenovo Tab 8 (2GB RAM + 16GB storage) running Android Lollipop 5+. (£60 - £70 at the time of purchase). We controlled for the differences in RAM by disabling all background activity on the Lenovo 7 tablets.

After the five days, when each participant returned their Cozmo robot and accompanying tablet, we measured various features of Cozmo’s interaction sessions with the participant. There were two ways this could be done:

- The SaveData.json file
- The Cozmo usage log file

The SaveData.json files stored a snapshot of the latest status of the games and tasks with the Cozmo app. This is the app’s saving point as the user progresses with unlocking new features of the Cozmo robot. When the app was closed and reopened again, it would refer to this file to locate the restart point to load. This file gave information about the final point that the participant reached after the five days. But there was no indication of what happened during the five days. For example, what game the participants played, how long the interaction session lasted, or which emotions Cozmo displayed. This more detailed information could be retrieved from the Cozmo app logs. These details were logged automatically by the app and sent off to third-party servers ^29^. Once these logs were sent, they are deleted from the device. We ensured the participant could not connect the device to any network other than the Cozmo’s wifi and accidently sent the logs to Anki before extraction. Since the devices were provided by us, we locked access to wi-fi settings and made sure that the device only connected to Cozmo’s wifi.

The SaveData.json files are usually found in the Android/data/com.anki.cozmo/files folder. The ongoing interaction and chronology of the games played and feature unlocked are available in the log file. This provides much richer information than the final snapshot of the app status. As such, we concentrate on the log files and do not look at SaveData.json for data collection. The case study showed us that indeed data extraction is possible and that the Cozmo robot is a viable system for embodied human-robot interaction studies.

### Step-by-step instructions

The steps describe below to extract information from the log files was developed in 2017–2019 with the Cozmo app version 1.5 (cozmo-anki-cozmo.353.apk). Further descriptions and code can be found at: https://github.com/cozmo4hri/Device_Setup ^30^.

***Step 1: Cozmo app and tablet setup***

When preparing the tablets for handout, ensure that

- Participants cannot start up other apps and use up the RAM
- Participants can only charge the tablet using the USB
- Participants cannot change the settings inadvertently
- Participants cannot connect to any other wifi network

If devices allow, create a separate participant user with no screen-lock. The main admin user needs to be locked using any available method (pin/pattern) which should not be disclosed to participants. Where it is not possible to have two separate users, using the inbuilt screen locks is not the solution as this will block access to Cozmo app too. Instead, use a separate Applocker software. This allows to selectively leave the Cozmo application unlocked while locking all other apps and settings. The Applocker****** should also be set to block options for installing and uninstalling all apps including itself. The only app that should be left for free access is the Cozmo application.

The device needs to be paired with the Cozmo robot to be handed out with it and set to automatically connect to Cozmo’s wifi. Active wifi searching must be disabled on the tablet to prevent it from connecting to any open wifi hotspots. Similarly, USB connection should be set to charge-only for the tablet. This prevents access to the file system or changing settings via a computer. The Applocker options for free access to device setting should also be blocked.

***Step 2: Setting/resetting the Cozmo app***

Before handing out the Cozmo robot to a new participant, any trace of previous use must be deleted. This guarantees that every participant gets the same start-point of interaction with Cozmo. We also set the initial registration of the app to a predefined lab-user so that participants could not enter sensitive information, like their birthdate, during the registration. The following steps achieve this:

1. Use the Cozmo apps inbuilt ‘Erase’ feature to delete and reset the Cozmo. This deletes any unlocked features and games but leaves the coins and sparks the last user earned.

2. We recommend keeping a copy of SaveData.json and SaveData.json.backup right after registering the Cozmo app. We overwrote the post-participation copies of SaveData.json and SaveData.json.backup with our initial copies. This achieved two things:
  ◦ It set the start point of the next load to right after the registration, so participants did not have to provide sensitive information
  ◦ It erases the sparks and coins earned, thus levelling the starting point for each participant.

3. Make sure all the app logs are deleted from the previous participant or extraction.
4. Reset the usb access mode to ‘charging only’ and lock wifi access to the Cozmo if these have been changed.

***Step 3: Cozmo app logs***

**Figure 2.**
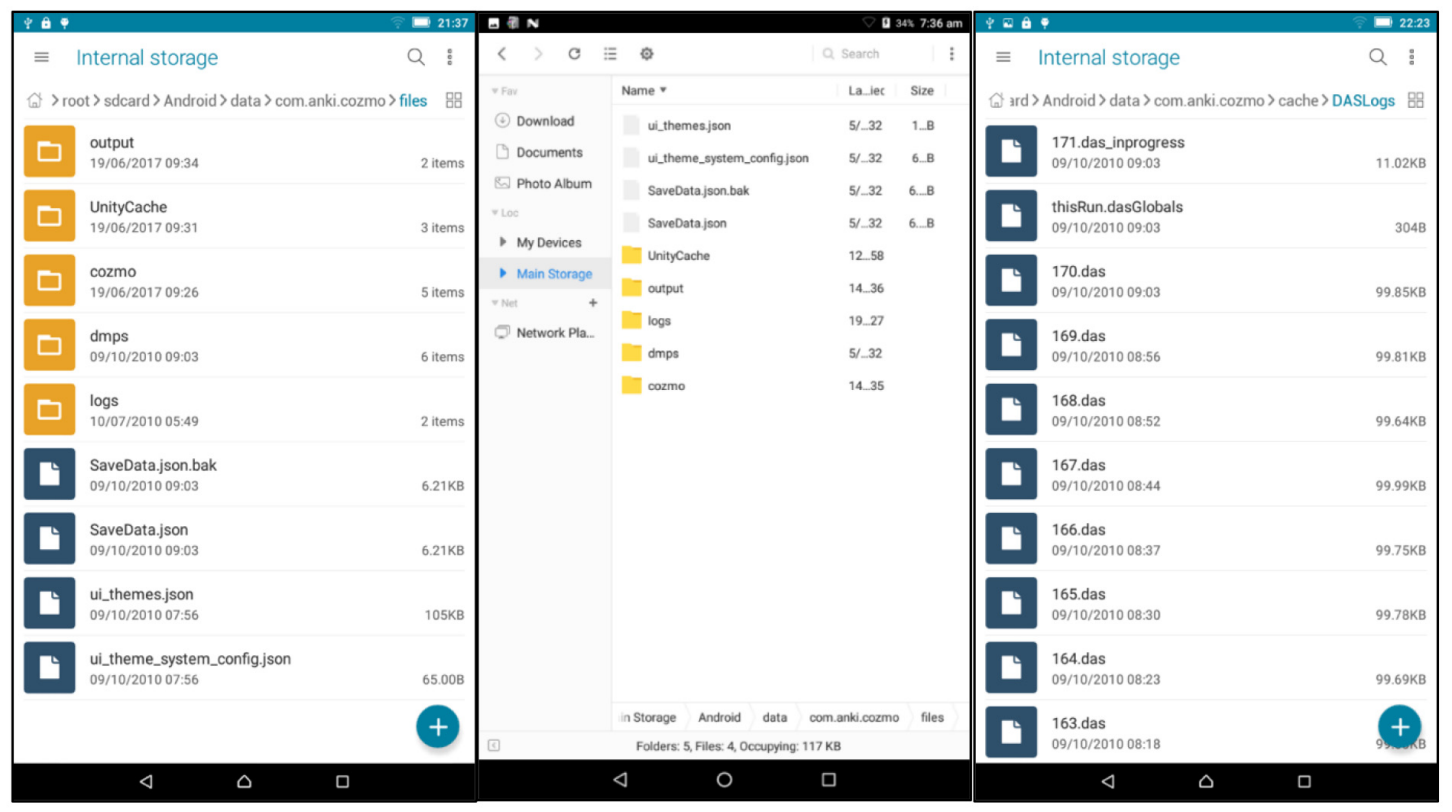
Log files storage. The Android/data/com.anki.cozmo/files folder containing SaveData.json in Lenovo Tab 7(left) and Lenovo Tab 8(middle), and the indexed .das files logging from Cozmo app (right).

Developers usually use application execution logs to monitor the health and chronology of event occurrences. These logs help with not only diagnosing issues with the app but also with improving performance by monitoring the application usage ^31^. With regards to the Cozmo robot and app, Anki logged events such as games and feature usage, scores, features unlocked, interactions, and the animations displayed by Cozmo. Anki avoided logging sensitive data that could be used to identify the user ^29^. Multiple app log files are written in each interaction session with Cozmo. An interaction session is the time when the app is started up and connected to the Cozmo robot to when the app is closed. The log files are written as indexed ‘.das’ files ( Figure 2).

To start a session with the Cozmo robot one must first setup the wi-fi on the Android devices to connect to the Cozmo’s wi-fi. Subsequently the Cozmo app must be started up and it will automatically connect to the Cozmo robot over this wi-fi. This is when the logging starts. The logging system looks for existing files in the directory and then initiates the current session log index from where it left off from the previous logging session files that are still in the directory. Each log file is 1KB in size. As the files are written, the index number is incremented, giving each file a unique name. When the session is completed by closing the app the lastUse.log is written. If at this point, the user disconnects from the Cozmo robot’s wi-fi connects to a different wi-fi for internet access, the log files are transferred to Anki’s Data Analytics server ^29^. Once a log is transferred to Anki, it gets deleted from the local Android file system. It is critical to take a copy of the files before the devices are connected to the internet to prevent such loss of data.

***Step 4: Extracting log files form the Androids***

The log files are stored in the default location of the filesystem where all application data are stored. This is the folder called ‘Android’ on its main storage or internal storage. To explore the Android’s file system, one can use the storage explorer in settings or install a file manager app.

 For us, the log files were in the folder **'**Android/data/cozmo.anki.cozmo/cache/DASLogs’. To move the files to a computer one needs to connect the tablet to the USB port with more than 'charging-only’ permission. In some cases, the Android folder is not visible on the computer depending on operating system and drivers. In such case moving the log files to the download folder is a possible workaround. Once log files are copied to the desired folder on the backup storage, they may be deleted from the Android device. Alternatively, the device may be reconnected to the internet for Anki’s collection process to start. Note, once the '.das’ log files are removed from the folder the Cozmo app will start reusing the indexes. So, a method is required for tracking the chronology of the extracted logs. This may be done by maintaining timestamped collection folders on the off-device storage each time the logs are extracted to it.

***Step 5: Log file entry***

**Figure 3.**
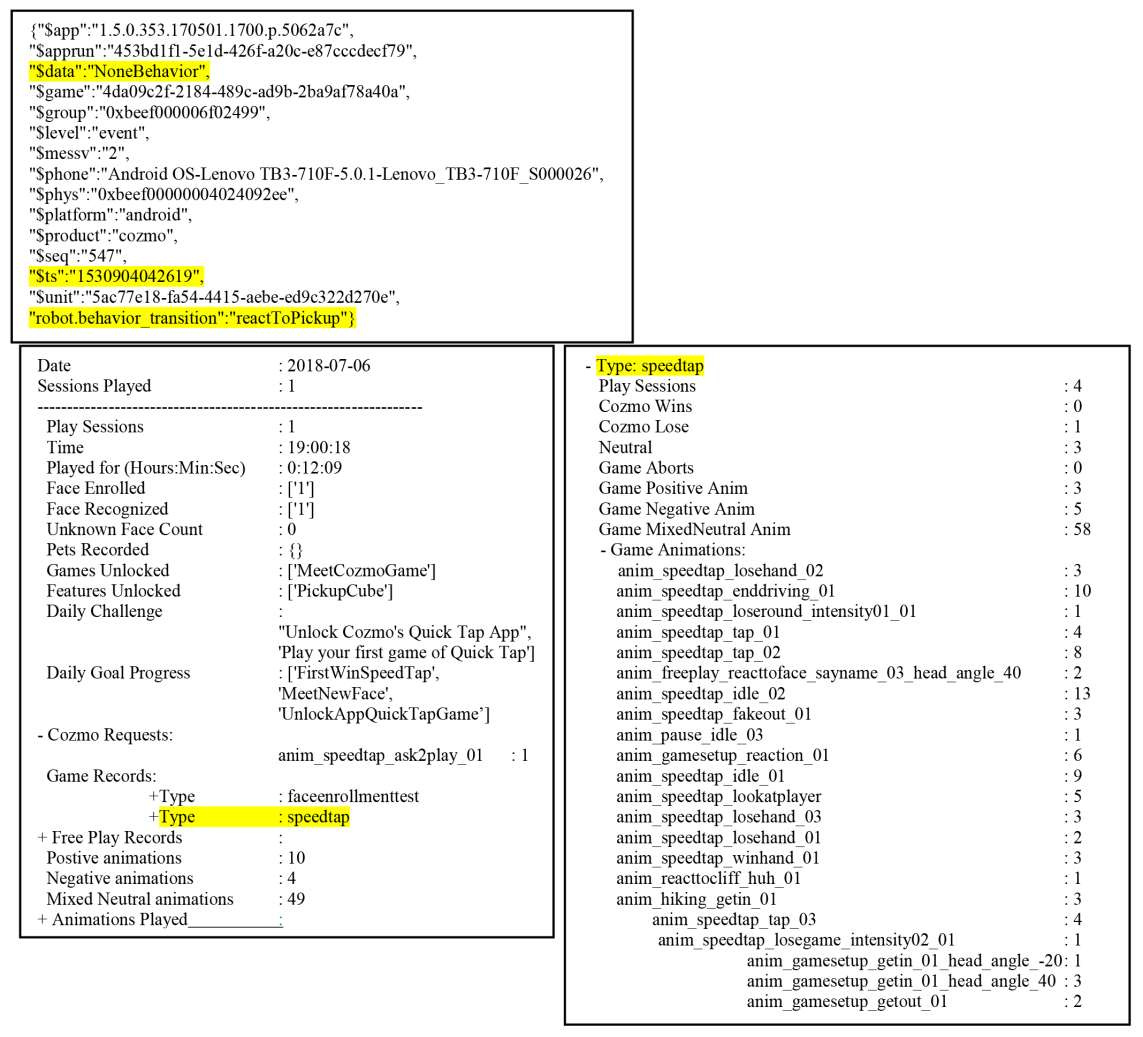
Interaction data example. A single log entry (top) and the output (bottom two panels). The general data extraction method identifies duration and details of play sessions and the games the user played (bottom left) and also the details of the scores and animations seen during each game that Cozmo played with user (bottom right).

Within each .das file the log entries are written in Java Script Object Notation (JSON) file format ^32^. Each line records the data as a collection of key/value pair. To parse the entries, we used a python program using a standard python json library. The individual keys against which the data was recorded were noted first. There were some familiar keys like those for recording the application ID, timestamp, and the device model. The last entry of each line was Anki’s in-house key-value pair that gave the kind of interaction data that we were after ( Figure 3 and Table 1).

**Table 1.** Example real world events mapped to the $custom (key:value) field from the point Cozmo wakes up to when it looks for a face and meets a new user.

Visual event	Log: $custom key	Log: $custom value
*Event: Cozmo wakes up*		
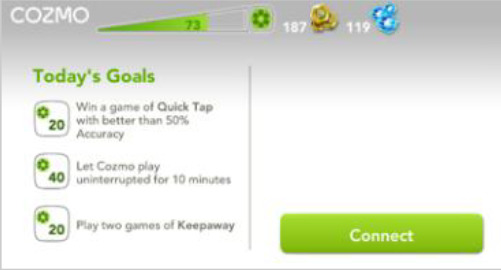	ui.button	connect_button
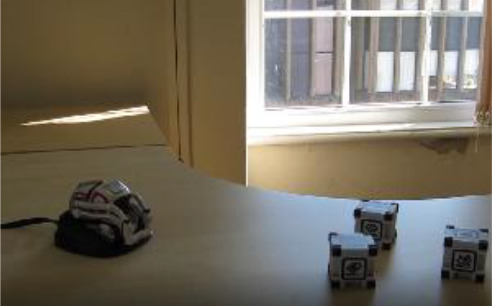	robot.behavior_transition	NULL
	robot.behavior_transition	NoneBehavior
	robot.handle_robot_set_head_id	0xbeef00000c508983
	robot.firmware_version	1981
	robot.object_located	Charger_Basic
	robot.on_charger	
	robot.game_unlock_status	DroneModeGame,KeepawayGame,MeetCozmoGame,MemoryMatchGame,QuickTapGame,CozmoSaysGame
	robot.spark_unlock_status	BuildPyramid,KnockOverThreeCubeStack,PickupCube,RollCube,StackTwoCubes,PounceOnMotionAction,PopAWheelieAction,Workout,FistBump,PeekABoo,
	robot.vision.loaded_face_enrollment_entry	3
	robot.vision.loaded_face_enrollment_entry	94
	robot.accessory_connection	0xe897627b,Block_LIGHTCUBE2
	robot.accessory_powerlevel	1 1.36V (1 lost)
	robot.accessory_connection	0xb972cb8b,Block_LIGHTCUBE3
	robot.accessory_powerlevel	2 1.42V (1 lost)
	robot.play_animation	anim_launch_wakeup_01
	robot.vision.image_quality	ImageQualityGood
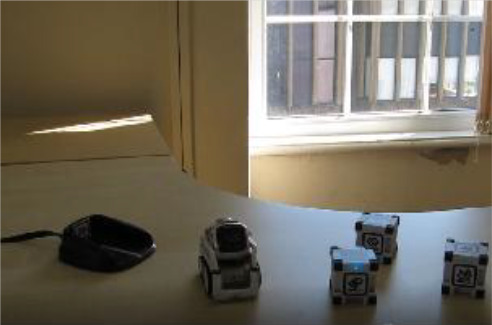	robot.mood_values	0,0,0,0.1,0,0,0,0,1,
	robot.behavior_transition	NULL
	robot.behavior_transition	findFaces_socialize
	robot.play_animation	anim_lookinplaceforfaces_keepalive_long_head_angle_40
*Event: Meet Cozmo*		
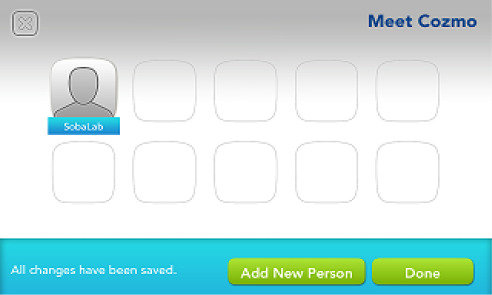	ui.slide.enter	face_list_slide
	robot.play_animation	anim_lookinplaceforfaces_keepalive_long_head_angle_40
	ui.button	add_new_person_button
	ui.slide.exit	face_list_slide
	ui.slide.enter	enter_new_name
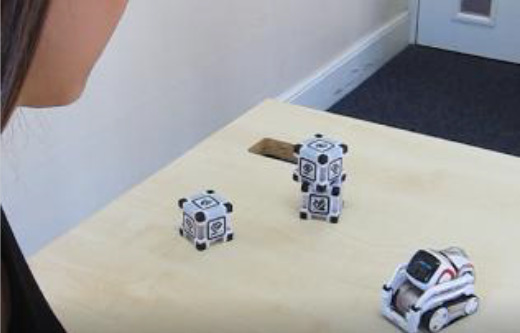	robot.behavior_transition	meetCozmo_interactWithFaces
	robot.mood_values	0.033265,0,0,-0.3,0,0,0.262434,0,0,
	robot.mood_values	0.0328382,0,0,-0.3,0,0,0.338734,0,0,
	robot.play_animation	anim_reacttoface_unidentified_03_head_angle_40
	robot.play_animation	id_motiontrack_turnsmall
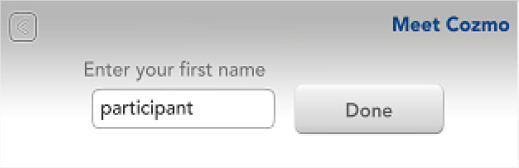	ui.button	enter_name_done
	ui.slide.exit	enter_new_name
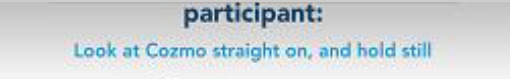	ui.button	enter_name_done
	ui.slide.exit	enter_new_name
	ui.modal.enter	face_enrollment_instructions
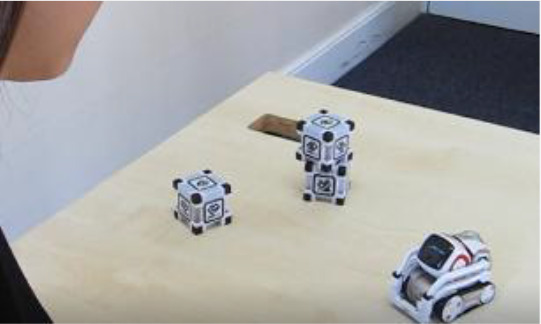	robot.behavior_transition	EnrollFace
	robot.mood_values	0.0303509,0,0,-0.3,0,0,0.418734,0,0,
	robot.mood_values	0.0286427,0,0,-0.3,0,0,0.498734,0,0,
	robot.play_animation	anim_meetcozmo_lookface_getin_head_angle_40
	robot.play_animation	anim_meetcozmo_lookface_interrupt
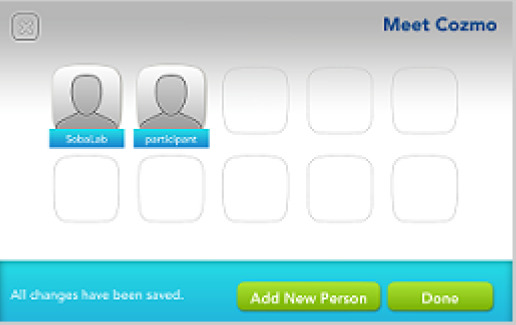	robot.face_enrollment	2
	robot.behavior_transition	NULL
	ui.modal.exit	face_enrollment_instructions
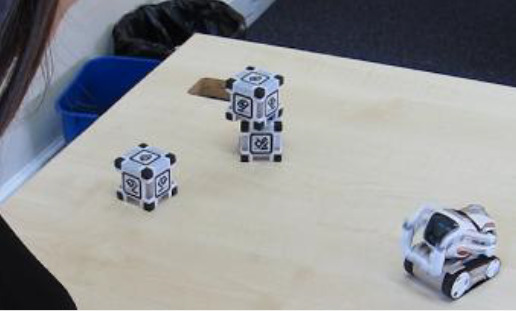	robot.behavior_transition	meetCozmo_findFaces_socialize
	robot.behavior_transition	meetCozmo_interactWithFaces

Photographs by Malte Heyen and Veronica Diveica, screenshots are taken from the Cozmo application version 1.5 developed by Anki, San Franscisco, CA, USA in 2017–2019.

 Of all the variables logged, we concentrated on the three entries that are highlighted. The ***$ts*** entry is the unix time stamp entry which gives the time at which the log was recorded. For Figure 3, the time stamp provided, 1530904042619, translates to: Friday, 6 July 2018 19:07:22.619 *.* The last entry of the log line is a custom (key : value) pair that gave a variety of information; the game played, user button selection on screen, the animation the robot played or the behaviour the robot chose to follow. They key entry in this case gave a hint as to what we were looking at. In Figure 3, the key is “robot.behaviour_transition” and the value is “reactToPickup”. The first part of the key before the ‘.’ also tells us which entry the log is about for e.g., robot, game, ui, app etc. This entry, we found, gave the maximum information about the state of the app and the robot. The ***$data*** entry was related to the custom entry mentioned above. In the case of “robot. behaviour_transition *”,* the data usually gives the previous behaviour it is transitioning from. In the example, it is reacting to a pick-up by the user, but was not exhibiting any previous behaviour hence the ‘None’ entry.

### Information stored in the log files

***General data extraction method***

Initially, we were interested in the general quality and quantity of the interaction with Cozmo robot over a five-day period. In our first long-term interaction study ^26^, we studied empathy towards a robot before and after the five days of socialisation. For this, the log extraction was meant to verify sufficient socialisation. We wanted to extract how many times and for how long participants interacted with the robot, and whether they were actively or passively interacting with the robot. We defined the ratio of self-initiation of the robot as the total number of requests made by the robot (e.g., ‘anim_rtpkeepaway_askforgame_01’) divided by the total behaviours of the robot. For this reason, we decided to extract all Cozmo’s animations that Cozmo displayed.

Our first challenge was to define the interaction session, as there were no explicit markers in the indexed log files. An interaction session is the time that spans when a participant switches on the robot to when they shut it down or lose connection to it. There were some gaps in the logs from time to time where nothing was logged. Our initial assumption was that 15 minutes or more of inactivity in the logs defined an inter-session interval. To understand the log, we collated each session data by interesting events that we identified from the custom entry key. See Table 2 for the events we extracted. The code for this can be found at: https://github.com/cozmo4hri/CozmoLogClean1.1 ^33^.

**Table 2.** Events extracted using the general data extraction method.

Interesting Event	Explanation
Date	The date the session(s) was logged on
Sessions Played	The Number of session(s) played on an given day
Play Sessions	Index of session being recorded
Time	Time of day when the session took place
Played for (Hours:Min:Sec)	How long the session lasted
Face Enrolled	Number of new faces enrolled with the robot
Face Recognized	Number of known faces the robot detected
Unknown Face Count	Number of unknown faces the robot detected
Pets Recorded	Number of Pets recorded by Cozmo
Games Unlocked	The games that were unlocked during the session
Features Unlocked	The features that were unlocked during the session
Daily Challenge	The daily challenge posed by the app
Daily Goal Progress	The progress made with respect to the daily challenges
Cozmo Requests	The number of ask or request animation Cozmo played to initiate interaction
Game Records	The section for details of games played with Cozmo in the session
Free Play Records	The activities that Cozmo was doing on its own
Positive animations	The record of animations with positive emotion words in its name
Negative animations	The record of animation with negative emotion words in the name
Mixed Neutral animations	The record of animations that were deemed mixed emotion or neutral emotion
Animations played	The record of all other animations

***Detailed data extraction method***

**Figure 4.**
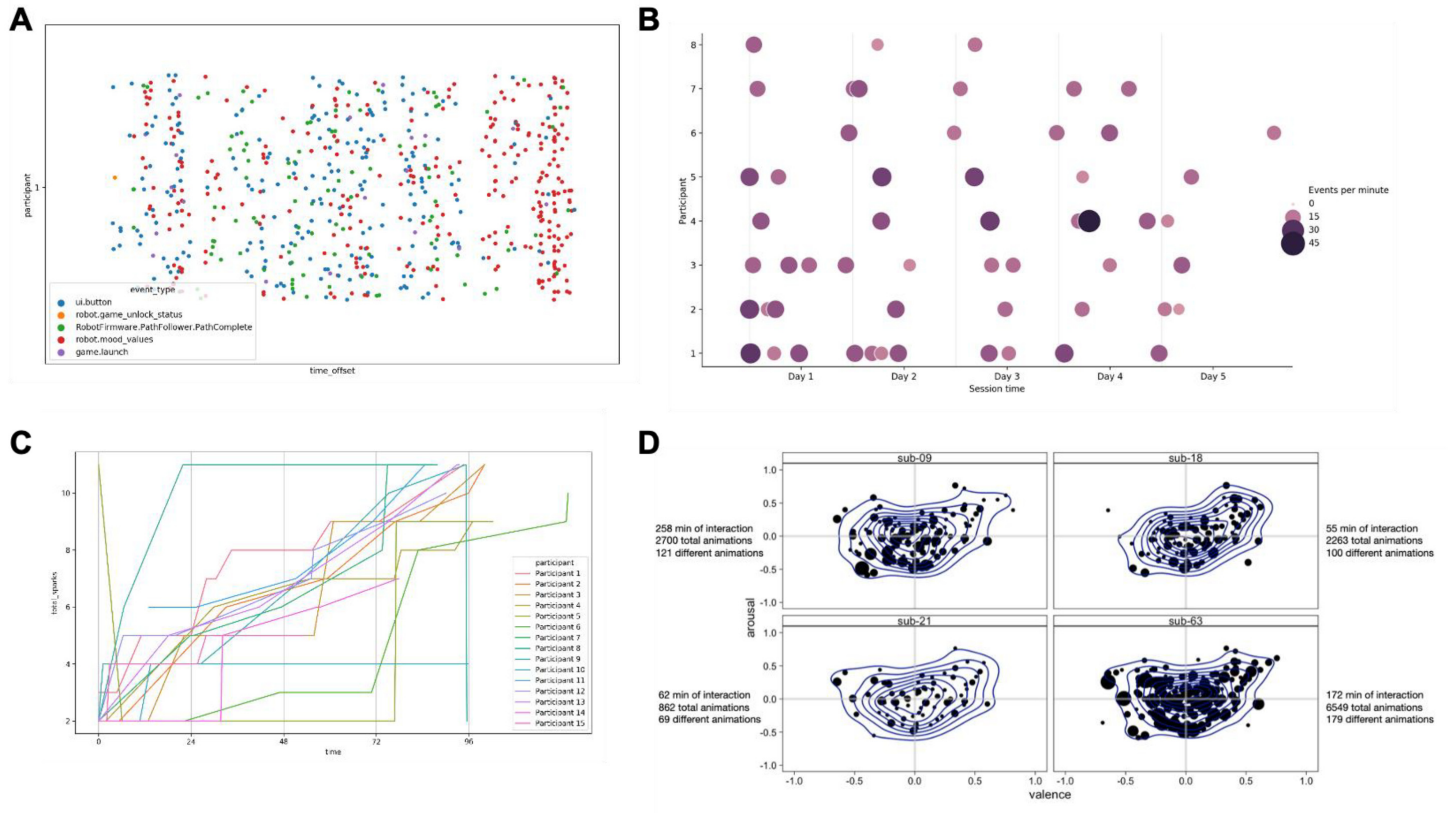
Detailed data extraction. **A**) Overview of a participant’s interaction with the Cozmo robot and app, **B**) a session engagement heatmap for one participant showing the events per minute and session duration, **C**) spark acquisition for several participants across time, and **D**) a data-driven overview of the emotional behaviour of the Cozmo robot during a five-day interaction period with the user. The combination of the data extracted from the log files (total duration, and total and number of different animations) and the emotional classification tool allows to establish individual behavioural profiles for the five-day interaction for each individual.

For developing further understanding of the user interaction we extracted the three fields of interest and the time stamp we identified for all log entry and visualised the data. The code for this can be found at: https://github.com/cozmo4hri/CozmoLogClean2.1 ^34^. We first verified our 15 minutes inter-session assumption. To validate this definition, we visualized the number of individual sessions we got by defining intersession log intervals going from one minute to 30 minutes. This allowed us to verify that the number of individual sessions did indeed stabilize when the intersession interval was defined as >15 minutes. Since our primary interest was to look at the quality of interaction that the participant had, we tried to visualize some key indicators for each participant These gave us an overview of whether the participant was interacting with the app through the user-interface (UI) screens or playing games and unlocking feature while interacting with the robots ( Figure 4A).

 To obtain an overview how long the participants engaged with the robots in each session over the five days we used a heatmap ( Figure 4B). This gave us an indicator if the session duration was declining over the five days. We also wanted to understand if the sessions themselves were busy in terms of the number of events that were being logged. A busier session would indicate a more active interaction. This could be cross-checked with how the participant progressed with the spark acquisition over the five days to check the engagement pattern ( Figure 4C). Combining the emotion classification data with the data extracted from the log files allows researchers to use a data-driven approach to map the emotional behaviour of the Cozmo robot during the interaction with the user ( Figure 4D).

### Emotion classification

In order to fully understand and reproduce one-off, long-term or longitudinal interactions with this autonomous robot, it is critical to assess not only the quantity of interaction time, but also details about the quality of the interaction. An important step toward achieving this is to map the behavioural repertoire of the robot, specifically the emotional content and value of its behaviours. As each behaviour of Cozmo is an execution of an animation, we focused our classification on individual animations. We mapped the behavioural repertoire of the robot using a two-step process. First, we extracted all the animations from the application version 1.5.0 and used the animation group name (e.g., ‘ask’, ‘badword’, ‘turtleroll’, ‘hiccup’) to subjectively classify the animation as either social or not social, and subsequently quantify the social animations as either a positive or negative emotion. We used this in a first study on the impact of socialising with the Cozmo robot on a distinct aspect of social cognition ^26^. Here, we built on this step by using a data-driven approach to classify the emotional content of these animations. This approach should provide researchers with a relatively unbiased and agnostic account of the behavioural repertoire of the robot. We video recorded the in-built animations of the Cozmo robot and asked independent raters to observe these videos and answer question related to the animations.

### Data collection

Participants (total *n* = 306; 123 women, 181 men, two unknown; aged between 18 and 71 years; mean age ± standard deviation 33.12 years ± 11.23) were recruited through Prolific ( www.prolific.co), provided active informed consent online, and were compensated with £0.50 upon completion. Only participants with an approval rate of 100 and who did not complete two experiments on the same topic (social perception of robots) from our group were able to participate.

As a proof-of-concept, we focussed on the animations for which the animation group name was assessed as social in the first step (348 animations out of the 790 animations in total) ^35^. In Experiment 1A, every participant ( *n* = 264) rated 10 videos that were selected from the animation database ^36^. The selection of which set of videos to show to each participant was based on an algorithm that kept track of the number of times each individual video had been rated in real time. Every time a new participant started the experiment, the videos that had been rated the least at that time were potential candidates for the new participant to see. This was done to ensure that by the end, each video would be rated a similar number of times. In an effort to reduce bias, videos were also selected so that each participant would see no more than one video from each animation group (e.g. ask, celebrate). As a result of balancing these two constraints, as well as great variation in the number of unique videos between the different animation groups, there was some variance in how often each video was actually rated. At the end of the experiment, each video was rated by at least six participants, while some videos were rated by up to 11 participants. The mean number of ratings per video was ~7.56. In Experiment 1B participants ( *n* = 42), who did not participate in Experiment 1A, rated the same 10 videos to test the variability of the ratings.

Participants were asked to rate each video on valence and arousal, as well as their confidence on these ratings. We provided a definition for valence (pleasantness of a given emotion, i.e. ranging from very positive/happy to very negative/unhappy) and arousal (excitation, intensity of a given emotion, i.e. ranging from active/excitable to passive/calm) ^37^. Participants were asked to select how they perceived the emotion displayed by the robot by selecting a position on a 2D grid, where the x-axis displayed the valence dimension (negative – positive) and the y-axis displayed the arousal dimension (relaxed/inactivated – stimulated/activated). Some videos showed the robot idle for a short amount of time, and in these cases, participants were asked to focus on the part of the video where the robot was active. Participants saw each video only once and were asked to indicate the confidence of their rating accordingly. After completion of the rating task, participants indicated how often they engaged with robots in daily life (on a scale from 1 (never) to 7 (daily). Total duration of the experiment was ~5 minutes.

## Results

**Figure 5.**
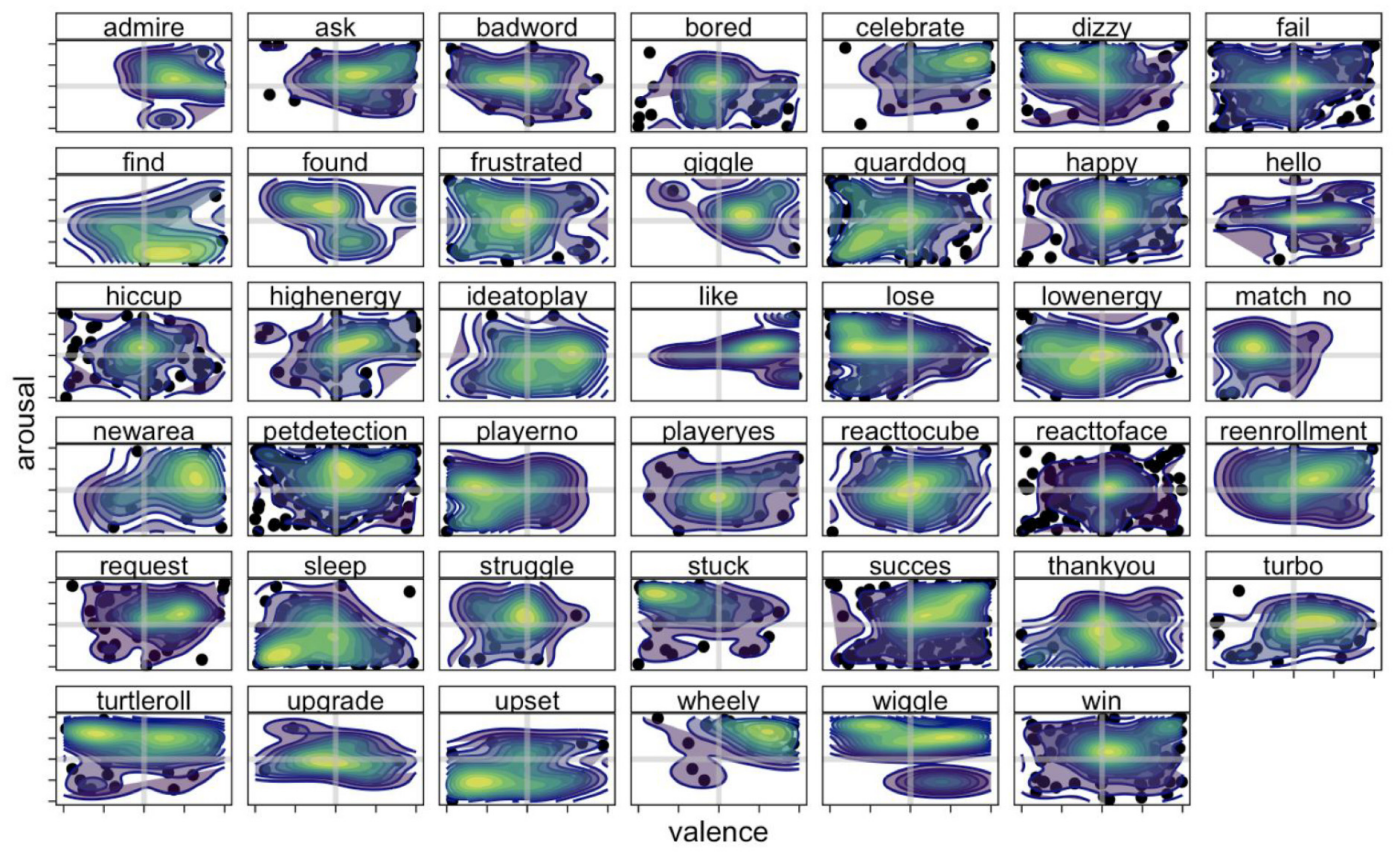
Valence and arousal ratings across animation groups (Experiment 1A). As can be seen from the heatmaps there is no one-on-one relation between the name of the animation group and perceived valence and arousal. For instance, while celebrate is seen a high arousing positive emotion, the animation groups of happy, fail, and win are not corresponding to their descriptors. A data-driven approach will prevent misclassification of the emotion and behaviour of the Cozmo robot. The x-axis shows the valence rating for each of the animations, ranging from -1 (negative) to 1 (positive), while the y-axis shows the arousal rating, ranging from -1 (relaxed/inactivated) to 1 (stimulated/activated). The heatmap represents both the density of the valence and arousal rating. Each dot represents a rating for an animation by a participant.

**Figure 6.**
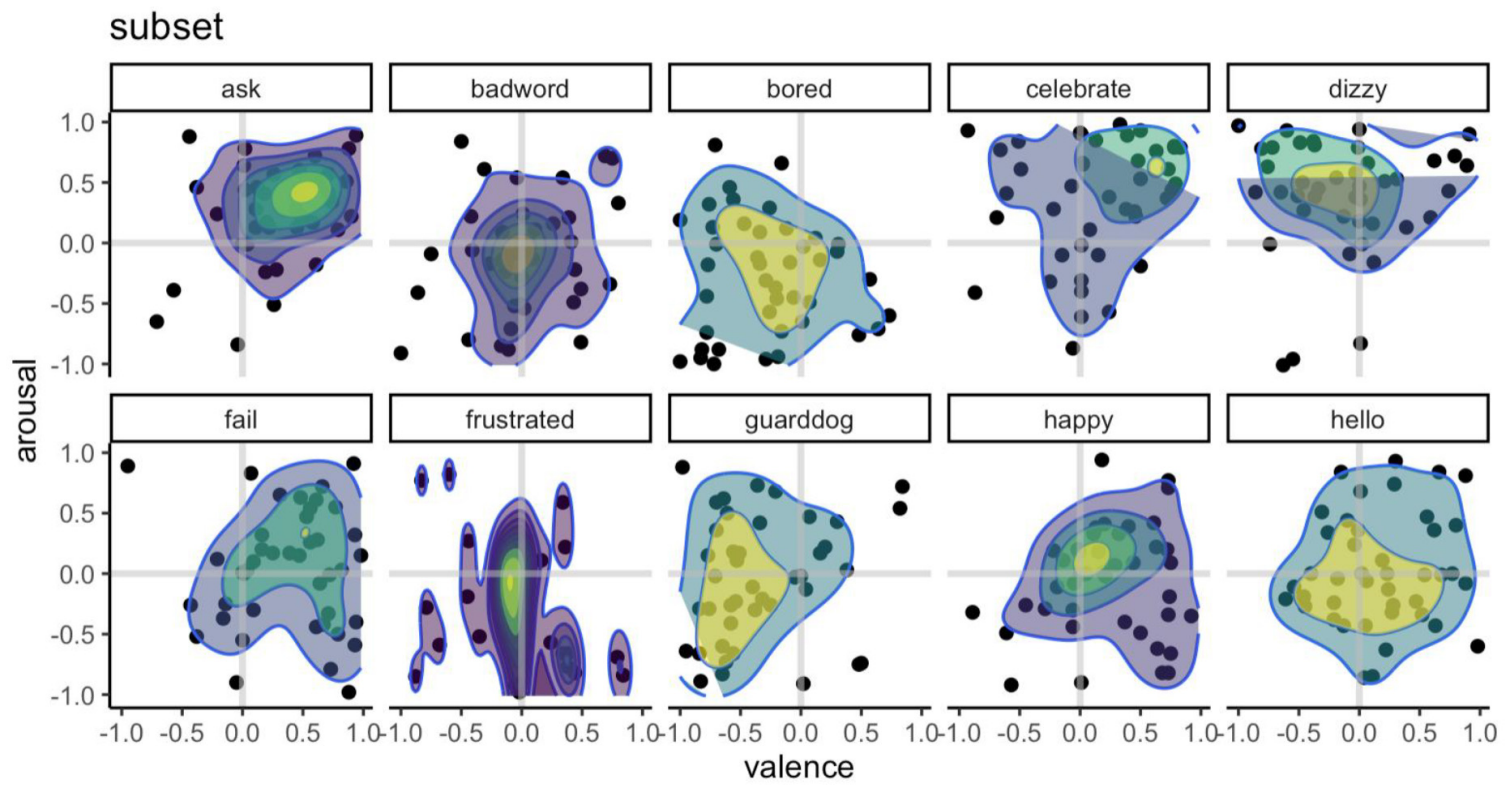
Valence and arousal ratings for a subset of the animations (Experiment 1B). While ratings vary across participants, the animations are associated with a distinct profile on the valence/arousal grid. The x-axis shows the valence rating for each of the animations, ranging from -1 (negative) to 1 (positive), while the y-axis shows the arousal rating, ranging from -1 (relaxed/inactivated) to 1 (stimulated/activated). The heatmap represents both the density of the valence and arousal rating. Each dot represents a rating for an animation by a participant.

Participants were confident in their rating of the videos, mean confidence ± standard deviation: 0.73 ± 0.21 (Experiment 1A); 0.70 ± 0.18 (Experiment 1B) on a scale from 0 to 1. Results showed that the animation groups show a distinct profile for valence and arousal ratings ( Figure 5). Some animations were rated as positive in valence and high arousing (e.g., 'celebrate’), while other animations are seen as positive in valence but low in arousal (e.g., 'find’). Similarly, several animation groups were viewed as negative in valence, but differed in level of arousal, ranging for low arousing (e.g., 'upset’) to high arousing negative animations (e.g., 'stuck’). It is of note that, on average, the animations centred around zero for both valence and arousal, suggesting that there is no clear affective label associated with the overall behaviour of the Cozmo robot. Ratings of arousal and valence did not correlate, *r*(262) = 0.08, 95% confidence interval [-0.04 , 0.20], *p* = .2, suggesting that animations could be mapped along two separate dimensions. Data from Experiment 1B showed that ratings across these dimensions vary slightly across participants, with standard deviations ranging from 0.38 to 0.51, with some animations (e.g., 'anim_meetcozmo_celebration’ from the 'celebrate’ group) occupy a smaller, more distinct, location on the quadrant than other animations (e.g., 'anim_pyramid_reacttocube_frustrated_low_01’ from the 'frustrated’ group; Figure 6). These findings emphasise the need for a validation procedure, such as the one employed here, that enables researchers to carefully select animations that convey the valence and arousal needed in a particular laboratory study, or to accurately map the behaviour of the robot in an interaction study.

**Figure 7.**
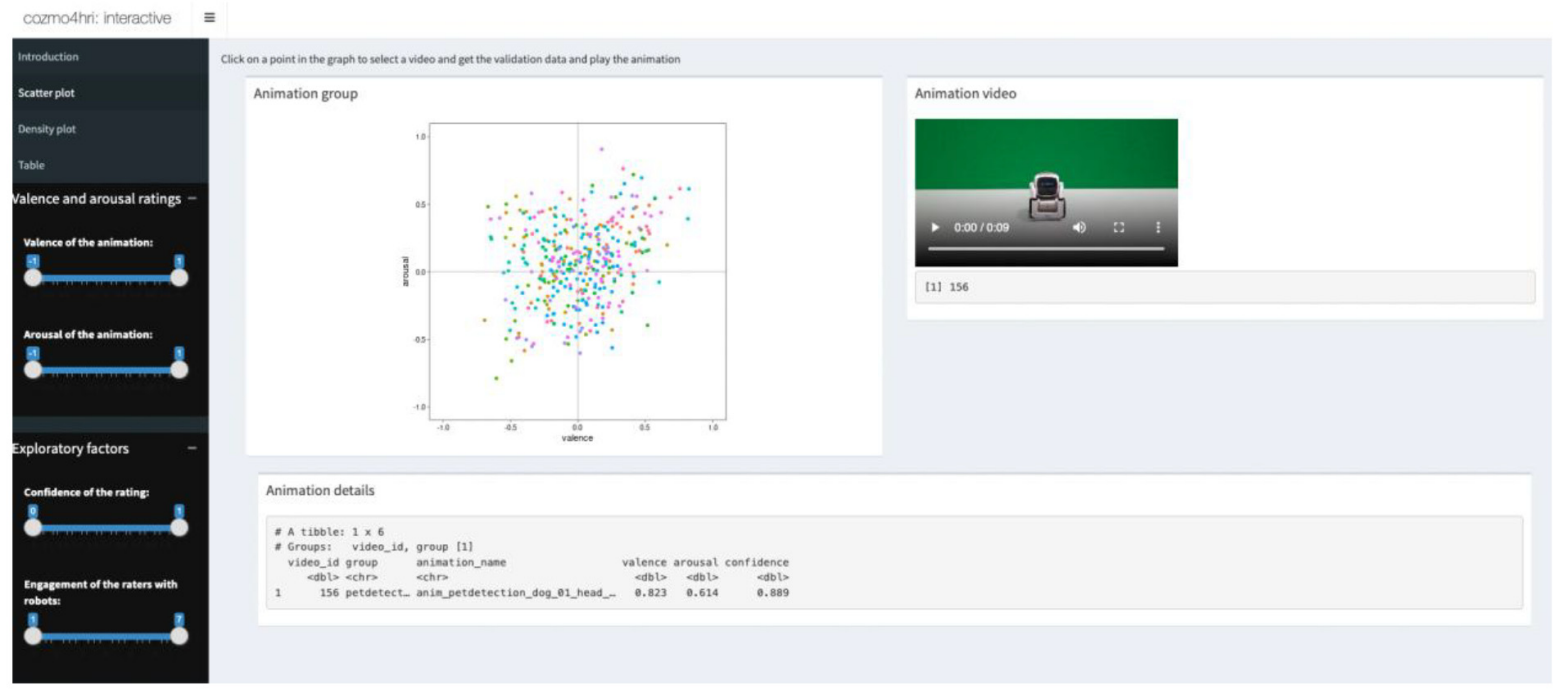
Introducing the interactive tool for emotion classification of Cozmo’s behaviour. Researchers can use this tool to get the validation data for an animation of the Cozmo robot, select and play animations based on valence and/or arousal or explore the impact of confidence and exposure to robots on these ratings. The interactive tool is publicly available at: https://shiny.psy.gla.ac.uk/ruudh/interactive-tool/.

To facilitate this, we created an interactive tool ^38^ using the Shiny package ^39^ based on the ratings of Experiments 1A ( Figure 7). This interactive application is publicly available and can be found here: https://shiny.psy.gla.ac.uk/ruudh/interactive-tool/. With this tool, researchers are able to select animations based on valence and/or arousal, as well as explore the impact of confidence of the ratings and exposure to robots. Researchers can also use the validation procedure and tool to validate other animations from future releases of Cozmo applications. In sum, we believe that this data-driven tool will help researchers make an informed decision of which animations to use in their study, but also provide a clear indication of the emotional content of the interaction between the robot and the user.

## Discussion

Here, we describe how a low-cost, easy-to-use, commercially available Cozmo robot can be used to study embodied interactions across settings and populations. In particular, we outlined how researchers can collect and extract data from this robotic platform to assess not only the quantity but also the quality of these interactions. Integrating this procedure with the interactive emotion classification tool allows researchers to employ a data-driven approach to human-robot interaction. We believe that these tools will provide the community with the necessary means to understand real interactions with social robots both within and beyond the research laboratory.

 We sketch only some of the several possibilities that these data provide the researcher. Beyond the duration or type of events, the log files recorded by the Cozmo application contain a rich set of variables that range from face recognition to daily goal progress. Such information can be combined with self-report, behavioural testing, neuroimaging, observational assessments by researchers, parents, or teachers, or other primary or secondary data sources. Above and beyond the validation of the animations that are displayed by the Cozmo robot during the interaction with the human user, these data can be used to select and display specific animations. We have successfully used this in studies employing one-off interaction games, by engineering emotions exhibited by Cozmo during various games manipulations ^19, 20^.

Several limitations must also be acknowledged in order to inform and improve future research efforts in this area. Critically, we only had access to the event logs that were provided by the inbuilt Cozmo app. At the moment, the user or researcher does not have access to the source code of the Cozmo robot. If this is possible in the future, this would allow researchers to enhance the inbuilt logs with other customised entries or activate parallel logging of events pertaining to the research question only. Similarly, the application log files are focused on the robot’s activity and do not give a detailed view of the participant or the environment of the interaction. While we are able to see when the participant engaged with the Cozmo application or when they were playing a game with Cozmo, currently no details are available on other participant or environmental factors, such as whether or not other people were in the room (that Cozmo didn’t recognise), or other agents or objects were vying with Cozmo for the participant’s attention. In order to establish ground truth, one could consider recording the interaction session or asking participants to video record their interaction sessions (with the necessary privacy considerations in place), and compare information gleaned from the log files with information available from full video recordings of an interaction.

Of course, the tools described present only one perspective for performing behavioural human-robot interaction research with the Cozmo robot. The Cozmo robot is increasingly used to study human-robot interaction with several researchers developing ways to employ this robotic platform as a research tool. For instance, McNeill and Kennington ^40^ described a different procedure to validate the animations of the Cozmo robot that uses both human raters as well as a neural network, and use this to engineer novel animations/behaviours. In addition, the tools described and shared in this article will benefit from ongoing developments and discussion in the field, and this is why we have openly shared them. The Comzo application does work with devices other than Android. The reason we looked at Android devises exclusively was due to availability, costs, and flexibility in terms of data extraction. Similarly, we froze the Cozmo application at version 1.5 to ensure consistency across our ongoing studies. We have made all the animations, tools, and scripts publicly available to allow researchers to verify, replicate, and extend our tools and pursue other questions. For instance, one limitation related to the validation tool that future research will need to address is the restrictive sample. Over the last years, culture has been acknowledged as a critical influence on how people perceive and interact with social robots ^41^. Incorporating different samples (across cultures, ages, education levels, etc), tools, perspectives, and approaches will enable researchers to harness the untapped potential of commercially available social robots as research tools.

## Data availability

### Underlying data

Zenodo: cozmo4hri/animations. https://doi.org/10.5281/zenodo.6627976 ^35^.

The project contains the following underlying data:

- /animation-set (348 movies files from 1.MOV.mp4 – 348.MOV.mp4 containing the recorded animations of the Cozmo robot).

Data are available under the terms of the Creative Commons Attribution 4.0 International license (CC-BY 4.0).

Zenodo: cozmo4hri/interactive-tool. https://doi.org/10.5281/zenodo.6627984 ^38^.

The project contains the following underlying data:

- fullset_shiny.csv (full data)

Data are available under the terms of the Creative Commons Zero "No rights reserved" data waiver (CC0 1.0 Public domain dedication). 

## Software availability

### Cozmo script

Source code available from: https://github.com/cozmo4hri/animations.

Archived source code at time of publication: https://doi.org/10.5281/zenodo.6627976.

License: GNU General Public License version 3

### Installer for version 1.5 of the Cozmo app

Source code available from: https://github.com/cozmo4hri/Device_Setup.

Archived source code at time of publication: https://doi.org/10.5281/zenodo.6627982.

License: GNU General Public License version 3

### General Cozmo Log cleaner

Source code available from: https://github.com/cozmo4hri/CozmoLogClean1.1

Archived source code at time of publication: https://doi.org/10.5281/zenodo.6627982.

License: GNU General Public License version 3

### Detailed Cozmo Log cleaner

Source code available from: https://github.com/cozmo4hri/CozmoLogClean2.1

Archived source code at time of publication: https://doi.org/10.5281/zenodo.6627980.

License: GNU General Public License version 3

### Emotion classification procedure

Source code available from: https://github.com/cozmo4hri/emotion-classification

Archived source code at time of publication: https://doi.org/10.5281/zenodo.6628025.

License: GNU General Public License version 3

### Interactive tool

Source code available from: https://github.com/cozmo4hri/interactive-tool

Archived source code at time of publication: https://doi.org/10.5281/zenodo.6627984.

License: Creative Commons Zero "No rights reserved" data waiver (CC0 1.0 Public domain dedication)
