# Supplementary material for: Tracking human interactions with a commercially-available robot over multiple days
Source: Open Res Eur. 2022 Aug 16;2:97. [Version 1] doi: 10.12688/openreseurope.14824.1 (PMC10445930; doi:10.12688/openreseurope.14824.1)
Supplement: Peer review history

# Peer review: Reviewer response for version 1

**DOI:** 10.21956/openreseurope.16012.r30623

**Published:** 2023-02-28

**Authors:** Eiichi Yoshida

**Affiliations:** 1 Department of Applied Electronics, Faculty of Advanced Engineering, Tokyo University of Science, Tokyo, Japan

This article provides a practical guideline for using a commercially-available small robot Cozmo for human-robot interactions, focusing on a ready-to-use set of tools allowing researchers to program their own long-term experiments of human-robot interactions. Especially, the classification of video animations is reported in terms of valence and arousal based on the experimental results from ratings of 300 participants.   I appreciate the authors' efforts for sharing the common tools that facilitates time-consuming organization of human-robot interaction experiments. Combined with the robot platform that is affordable, this initiative is certainly expected to lead to wide participation into human-robot interaction research from wider areas and then to accelerate the research by forming a community and accumulating experimental data. I think the description of the software structure and usage is sufficiently clear and pedagogic. As noted above, the interactive tool for emotion classification of Cozmo’s behavior (Fig. 7) is a good contribution to fast startup of experiments.   As I am not really from the domain of the cognitive human-robot interaction, rather from pHRI, I may say something out of scope, but I suggest the following enhancements in the article as possible improvements, The authors probably meant the article towards the researchers already working on human-robot interactions. I understand this, but the paper would have broader readership from people in different disciplines if some more introductory presentations are added. For instance, some concrete exemplar experiments and expected outcomes by such experimental setup. In Fig. 1, there are some pictures of example of interactions, but I could not get really get what kind of interactions are induced by stacking cubes or playing games, what kind of psychological or social effects can be investigated by such interactions, etc. Some illustrative explanations on this point would be helpful. I also have another question about observing human behaviors. I understand the history of the robot behaviors have been recorded and this is very useful, but I think it is also important to measure the reaction of interacting persons in more detail. I suppose video recording of interacting human is of particular interest. While there may be of course issues related to privacy or individual information protection, by applying necessary measures exploitation of the data could have potentially a great impact on the research on understanding human status and synthesis of robot behaviors. If the robot can optionally have some other sensors like infrared detectors, it could also be useful.

Are sufficient details provided to allow replication of the method development and its use by others?

Yes

Is the description of the method technically sound?

Partly

Are the conclusions about the method and its performance adequately supported by the findings presented in the article?

Yes

Is the rationale for developing the new method (or application) clearly explained

No

If any results are presented, are all the source data underlying the results available to ensure full reproducibility?

No source data required

Reviewer Expertise:

NA

I confirm that I have read this submission and believe that I have an appropriate level of expertise to confirm that it is of an acceptable scientific standard.

# Peer review: Reviewer response for version 1

**DOI:** 10.21956/openreseurope.16012.r30622

**Published:** 2023-02-13

**Authors:** Hoang-Long Cao

**Affiliations:** 1 Brussels Human Robotics Research Center, Flanders Make, Vrije Universiteit Brussel, Brussels, Belgium

The manuscript presents an off-the-shelf method using a commercially available robot platform Cozmo to collect data in situ in real-world human-robot interaction. A step-by-step tutorial is presented allowing other researchers to replicate the work. Interaction data can be extracted to analyze the psychological consequences of long-term interaction (valence and arousal).

 Since most HRI studies are conducted in a lab-based environment, I appreciate the authors' effort to make this solution available to study HRI "in the wild".

 The authors listed several robot platforms that are frequently used in HRI studies (e.g. NAO, Pepper, Miro). I suggest the authors review a bit more platforms especially smaller-size robots (e.g. Keepon, Kirobo) that are more comparable in size with Cozmo.

 The level of anthropomorphism of Cozmo is low compared to other robot platforms used in HRI studies. A bit more explanation is needed about how this platform is suitable for HRI studies (for example the way of expressing human-like emotions).

Are sufficient details provided to allow replication of the method development and its use by others?

Yes

Is the description of the method technically sound?

Yes

Are the conclusions about the method and its performance adequately supported by the findings presented in the article?

Yes

Is the rationale for developing the new method (or application) clearly explained

Yes

If any results are presented, are all the source data underlying the results available to ensure full reproducibility?

Yes

Reviewer Expertise:

Social robotics, Human-robot interaction

I confirm that I have read this submission and believe that I have an appropriate level of expertise to confirm that it is of an acceptable scientific standard, however I have significant reservations, as outlined above.

# Peer review: Reviewer response for version 1

**DOI:** 10.21956/openreseurope.16012.r30072

**Published:** 2022-10-07

**Authors:** Merel Keijsers

**Affiliations:** 1 John Cabot University, Rome, Italy

The current paper is methodological in nature and discusses the use of an affordable off-the-shelf robot in psychological research. It focuses on two main points: how to access logged data on the robot that indicates the amount and type of interactions it has had, and to what extent the robot's pre-programmed expressions are universally interpreted as what they intended to convey.

 Considering how this robot is popular, comparatively cheap, has been used in previous research and already has some methodological papers written on how to modify its behaviour for research purposes, I would say the current paper is both timely and important. Furthermore, I appreciate how the authors looked at the human-robot interaction from two sides, highlighting both how data on the robot's behaviour (which is often evoked by a human and thus can inform us too on the human behaviour towards the robot) can be extracted, and giving warning on whether or not this behaviour will be interpreted the way it was intended to, thus identifying an important limitation to interpreting such extracted data carelessly.

 I have a few minor remarks that I believe should be addressed before publication. The authors argue that embodied HRI is vital and seem to imply that this is specifically the case for specific kinds of research. While I do not necessarily disagree, I would like to see some citations here. To my understanding and experience, at least when it comes to mind attribution to robots the issue is not so much physical presence but rather how interactive the robot is. A virtually presented robot that someone can interact with can be quite telling for cognitive attributions still.

 Furthermore, on page 6 under bullet point 1 the authors suddenly start talking about 'sparks and coins'. From the context I understand that those are some forms of counters that keep track of the kinds of interactions the human engaged in with the robot, but I'd appreciate a more precise introduction to the concept.

 Figure 4B has unlabelled cut-off points for the days, which means that as a naive reader I understand that most people interacted with their Cozmo between midnight and early morning?

 The results from the Valence/Arousal study are interesting and a little concerning; I do wonder if those behaviours would be more readily interpreted within context. I was reminded of this paper ^1^ that found that body language, not facial expressions allow people to tell whether an athlete had just won or lost. Of course, the body language of the robot was visible in the presented videos, but the expression of the Cozmo is more limited than that of humans, which means that we'd rely more on knowledge other than direct behaviour (what happened just before this emotional expression?) to interpret it.

 This leaves the original point of ambiguity of the expressions unchanged, but in real HRI those behaviours won't occur in a vacuum.

 Other than that, I am a fan of this submission.

Are sufficient details provided to allow replication of the method development and its use by others?

Yes

Is the description of the method technically sound?

Yes

Are the conclusions about the method and its performance adequately supported by the findings presented in the article?

Yes

Is the rationale for developing the new method (or application) clearly explained

Partly

If any results are presented, are all the source data underlying the results available to ensure full reproducibility?

Yes

Reviewer Expertise:

Social robotics, specifically mind attribution to robots and its consequences for human cognition, affection, and behaviour

I confirm that I have read this submission and believe that I have an appropriate level of expertise to confirm that it is of an acceptable scientific standard, however I have significant reservations, as outlined above.

## References

[1] : Body cues, not facial expressions, discriminate between intense positive and negative emotions. *Science* .2012;338(6111) : 10.1126/science.1224313 1225-9 10.1126/science.1224313 23197536
